# Personalized anti-inflammatory diets for allergic and skin disorders

**DOI:** 10.1186/1878-5085-5-S1-A160

**Published:** 2014-02-11

**Authors:** John G Ionescu

**Affiliations:** 1 Research Department of the Spezialklinik Neukirchen, Germany; Donau-University Krems, Austria

## 

Our experience in the treatment of over 20,000 atopic eczema, urticaria and psoriasis patients shows that besides allergic reactions to foods an increasing number of pseudoallergic reactions caused by toxic-irritative pollutants (formaldehyde, exhaust particles, additive-rich food, nicotine, wood preservatives, pesticides, heavy metals) are responsible for the inflammatory process behind the complex symptoms. Intrauterine and postnatal environmental influences were also reported. The routine analysis of specific IgE- and IgG4-factors in our atopic patients by means of ELISA-assays after challenge meals (CM) showed an increased frequency of specific IgE- and IgG4-antibodies after repeated CM. In 60% of the patients, we simultaneously found raised concentrations of the IgE- and IgG-CIC (p< 0.005) responsible for the delayed allergic reactions in the same group. Further inflammation markers like acute phase proteins (α1–antitrypsin, α2–makroglobulin, haptoglobin and caeruloplasmin) showed a surprisingly rapid increase after CM (p< 0.01) in the atopic group; by contrast the control samples remained in the normal range. Serum histamine levels (RIA-Test) also showed a significant increase 1/2 hour after CM and after individual oral provocation with lactose, sucrose, tyramine, serotonine or phenylethylamine, respectively. The carbohydrate intolerance reactions (H2-test) were in good correlation to the significantly lowered disaccharidase activites (lactase, sucrase) in the gut of atopic eczema patients (p< 0.001). This was closely related to chronic intestinal dysbiosis with toxic microbial breakdown products (alcohols, aldehydes, phenols, diamines) leading to an increased intestinal permeability, histamine release and impairment of liver detox functions. On the other side, we found both in atopic eczema and in psoriasis patients pseudoallergic reactions against biogenic amines and constantly raised serum histamine levels (also in fasted patients), suggesting an inhibition of catabolic enzymes (MAO, DAO, NMT). Previously published results from our laboratory showed significantly reduced DAO (p< 0.001) and Type B MAO- (p< 0.05) activities in thrombocyte-rich plasma of atopic eczema and psoriasis patients explaining their intolerance reactions to histamine, tyramine and octopamine rich foods. Last, but not least, the chronic increased levels of free radicals in whole blood and plasma of all patient groups showed significant changes after oral intake of different food homogenates/ juices dependent on their ROS-blocking or ROS-increasing effects (enhanced chemoluminescence test). Our current research shows that appropriate combinations of hypoallergenic protein hydrolysates with particular sugar alcohols and omega-3 fatty acids (EQUIDERM PLUS®) are dramatically enhancing the anti-inflammatory and free radical quenching properties of the mixture, demonstrating an excellent compliance and therapy efficiency in atopic, psoriasis and geriatric patients. With the emergence of affordable microarray gene expression profiling methods, the opportunity arises to *ex-vivo* test certain combinations for their silencing ability on inflammatory genes before administering the nutraceutical to the patient (nutrigenomic evaluation). The input of the above mentioned nutritional data in a computer supported, individual rotation diet plan (FOOD ALLERGY CONTROL®) ensures a gradual improvement of the symptoms and stabilization of the clinical course during and after therapy.

